# The incidence of avoidable healthcare-associated harm in prisons in England: a retrospective case note review

**DOI:** 10.1136/bmjqs-2025-019935

**Published:** 2026-05-07

**Authors:** Isobel Joy McFadzean, Saied Ibrahim, Verity Wainwright, Maria Panagioti, Melanie Jordan, Sandra Flynn, Richard N Keers, Tim Millar, Darren M Ashcroft, Adrian Edwards, Anthony J Avery, Jenny Shaw, Andrew Carson-Stevens

**Affiliations:** 1 Division of Population Medicine, Cardiff University, Cardiff, UK; 2 National Confidential Inquiry into Suicide and Safety in Mental Health (NCISH), Division of Psychology and Mental Health, The University of Manchester, Manchester, UK; 3 Division of Psychology and Mental Health, Centre for Mental Health and Safety, The University of Manchester, Manchester, UK; 4 Division of Population Health, Health Services Research and Primary Care, The University of Manchester, Manchester, UK; 5 NIHR Greater Manchester Patient Safety Research Collaboration, Manchester, UK; 6 School of Sociology and Social Policy, The University of Nottingham, Nottingham, UK; 7 Centre for Pharmacoepidemiology and Drug Safety, Division of Pharmacy and Optometry, The University of Manchester, Manchester, UK; 8 School of Health Sciences, Faculty of Biology Medicine and Health, The University of Manchester, Manchester, UK; 9 PRIME Centre Wales, Division of Population Medicine, Cardiff University, Cardiff, UK; 10 Centre for Academic Primary Care, The University of Nottingham, Nottingham, UK

**Keywords:** Patient Safety, Epidemiology, Chart review methodologies

## Abstract

**Objective:**

To estimate the incidence of avoidable healthcare-associated harm for prisoners in England.

**Design:**

A retrospective cross-sectional case note review of prisoner healthcare records.

**Setting:**

18 prisons in England were purposively sampled for maximum variation of characteristics based on prison category (open, local, training, high security and female), type (publicly and privately run) and population size.

**Population:**

After screening 15 027 prisoner records, two cohorts were selected: a sample of 6294 ‘enhanced risk’ prisoners and a random sample of 853 prisoners not included in the enhanced risk sample (n=7147).

**Main outcome measures:**

The primary outcome was the incidence of patient harm per 100 000 patient-years, judged at least probably avoidable. The secondary outcome was the incidence of patient harm judged at least possibly avoidable. Cases of avoidable harm were characterised in terms of patient impact, known as patient outcome(s), and the severity of harm experienced.

**Results:**

Within 18 prisons**,** 247 cases of avoidable harm were experienced by 244 prisoners and were identified from 7147 patient records. The incidence of avoidable harm was 2241.4 (95% CI 1970.5 to 2539.0) per 100 000 patient-years, and this rate could be as high as 3412.0 (95% CI 3075.8 to 3774.9) based on the records screened during this study. Most patient outcomes involved prisoners experiencing discomfort and pain (99/247, 40.1%) and delays receiving appropriate healthcare management or assessment (91/247, 36.8%). The identified cases of avoidable healthcare-associated harm for prisoners resulted mainly in moderate harm severity (157/247 cases, 63.6%), followed by severe harm (27, 10.9%) and death (27, 10.9%).

**Conclusions:**

Compared with community settings, people in prison experience a 41–67 times greater risk of avoidable significant healthcare-associated harm. This stark disparity underscores the urgent need for government and policy action. Delivering safe, equitable healthcare in secure environments remains a major challenge that demands focused attention.

**WHAT IS ALREADY KNOWN ON THIS TOPIC**

- Prisons are a challenging environment to provide care, exacerbated by the complex needs of the population.

**WHAT THIS STUDY ADDS**

- Estimates of avoidable harm in prison healthcare substantially exceed those reported in community settings.

**HOW THIS STUDY MIGHT AFFECT RESEARCH, PRACTICE OR POLICY**

- An integrated focus on healthcare provision, particularly concerning prisoner physical and mental health, is needed to address the systemic failures contributing to harm.

## Introduction

 There is a burden of patient harm globally, impacting health expenditure, patients, their families and the public. Patient harm affects perceptions of medical care and impacts morbidity and mortality worldwide.^1^ Approximately one in 20 patients experience avoidable healthcare-associated harm,^2^ with the World Health Organization (WHO), within their ‘Global Patient Safety Action Plan’, advocating for safety for every patient, every time.^1^

In 2024, nearly 100 000 people resided across prison and youth offender detention services across England and Wales (i.e, prisoners).^3^ Prisoners have individual medical needs and require access to healthcare services in and outside of prisons. Prison populations are ageing, with higher rates of morbidity and premature mortality, often living in areas of deprivation with higher rates of poverty and lower levels of education,^4^ which is a public health concern.^5 6^ Studies have highlighted problems with prison healthcare delivery, accessing healthcare appointments and healthcare professionals.^7 8^ Concerns have also been raised in investigations by the Health Services Safety Investigations Body in England, identifying challenges with emergency care provision and managing long-term conditions.^9 10^ Such inquiries and analysis of incident reports to assess patient safety^7 11 12^ provide insights into prison healthcare but cannot quantify harm frequencies.

The Department of Health and Social Care took over healthcare provision in prisons in England and Wales in 2006 to support care ‘equivalence’, that is, ‘staff, resources and facilities of at least the same standard as those available in the community’.^13^ However, healthcare delivery in prisons can be suboptimal and inequitable.^6 14 15^ Prison overcrowding, understaffing and increasing rates of self-harm and substance use among this group have prompted renewed calls for improvement,^16–18^ with equivalence difficult to achieve. While the issues compromising the quality and safety of prison healthcare are acknowledged, there is little research into understanding the frequency and nature of these problems, their avoidability and how to mitigate unsafe care. Given the vulnerabilities of the prison population and the challenges of delivering care in prisons, understanding and addressing patient harm in this context is urgent and essential for improving equitable healthcare provision. This study aimed to examine the incidence and severity of avoidable healthcare-associated harm in prisons in England.

The study objectives were:

- To estimate the incidence of avoidable healthcare-associated harm for prisoners.
- To quantify and classify the severity of avoidable patient harm.
- To examine prison-level characteristics such as population size, security category and levels of deprivation that may be associated with avoidable harm.

## Methods

### Study setting

As per our protocol,^19^ 18 prisons in England were purposively sampled for characteristic variation by prison category (open, local, training, high security, female), type (publicly and privately run) and population size.^20^ Prisons were invited if they provided Governor approval, on-site healthcare services to prisoners and granted remote access to the electronic health record system SystmOne.^21^

### Study design

This is a large-scale retrospective cross-sectional study involving a two-stage case note review of healthcare received by prisoners over a 12-month census period.^19^ Examining healthcare records to assess care quality and safety can inform epidemiological estimates of healthcare-associated harm^22^ and whether it was avoidable. This has been used extensively in healthcare,^2^ including primary care,^23^ to direct improvement efforts.

### Population

The medical records of all prisoners present in the prison on the census day (online supplemental table 2) were screened to identify criteria which we hypothesised would increase their risk of harm (online supplemental table 3),^19^ and those meeting these criteria were named the ‘enhanced sample’.

The records of prisoners on remand who had died during the census period were also screened. This was because we had no way to calculate a possible release date or anticipate the outcome of hearings, and therefore whether they would still have been in the prison on the census day. Sentenced prisoners who had died were also included, depending on their expected prison duration.^19^

Subsequently, the medical records for two prisoner samples were included to determine evidence of healthcare-associated harm within a formal review: a sample of ‘enhanced’ risk prisoners considered to be at most risk of healthcare-associated harm, for example, with multimorbidity^19^; and a 10% random sample of prisoners excluded from the enhanced risk sample, known as the ‘outsample’. These samples were combined to estimate the incidence of avoidable harm, ‘per 100 000 patient-years’.^19^ The remaining records, which had been screened by nurses/doctors but did not undergo the case note review by general practitioners (GPs), were included within our overall denominator when estimating incidence rates of harm.

### Data screening

Screening of electronic primary care medical records, to determine whether the enhanced criteria were present, was undertaken independently by recruited nurses/doctors by reviewing records for up to 12 months before the census day (fewer if the prisoner resided for shorter durations). The denominator for the main outcome measure was the number of months of data for each prisoner, calculated overall as ‘patient-years’.

### Case note review

GPs were recruited to undertake the review of medical records, appraise their content and identify and describe evidence of avoidable healthcare-associated harm, written in a free-text narrative.^19^

### Training

Prior to conducting screening and reviews, the nurses, doctors and GPs completed a blended learning programme. This included asynchronous pre-recorded lectures and panel discussions with prison-based healthcare professionals. The programme covered: (1) defining their roles within the study, Human Factors in healthcare and principles of patient safety incident analysis; (2) descriptions of prison healthcare in the United Kingdom (UK), comparisons between prison and community-based primary care, and orientation to key documentation; and (3) example scenarios (based on real cases) of prison patient safety incidents.

### Harm

Patient safety incidents identified by reviewers were defined as ‘any unintentional or unexpected event, that led to harm, for one or more prisoners whilst receiving healthcare,’ adapted from the National Health Service (NHS) definition.^24^ This definition reflected acts of commission and/or omission^24^ and included both psychological and physical harm outcomes.^25^

Within the training, reviewers were trained to include all reports with a suspicion of harm (patient safety incidents) to ensure that harmful cases were not missed. A minimum of 10% of medical records within each prison were independently double-reviewed. If both GPs submitted the same incident, cases with the greatest detail were included for analysis to prevent incident double counting. Harms were included when they occurred during the prisoner’s time in prison and related to primary, secondary or tertiary healthcare. All cases submitted were then appraised by the patient safety experts within the study team (IJM and AC-S).

### Avoidability

Following preliminary work to define avoidable healthcare-associated harm within prisons,^11^ GP reviewers were asked to judge the ‘avoidability’ of harm on a six-point scale ranging from ‘definitely unavoidable’ to ‘definitely avoidable’ (see online supplemental table 1).

To clearly highlight the nuances of the prison environment, cases of healthcare-associated harm were judged against this scale in two ways:

(*Tier 1*): *equivalence with* *community* *care*—appraisal of whether more could have been done, as if care were delivered in the community (i.e, equivalence of care to achieve equitable health outcomes). This judgement was made without consideration of caveats introduced by the prison regime, system and environment.

(*Tier 2*): *accounting for the* *prison* *regime*—with prison-experienced healthcare professional input during analysis, further appraisal was made regarding whether the prison could have done more. This included considering restrictions imposed by the prison regime to identify aspects of the prison environment which may contribute to harm (e.g. resources, service availability or lack of prisoner autonomy to coordinate appointments).

## Analysis

### Primary and secondary outcomes

The primary outcome was the incidence of patient harm judged at least probably avoidable (cases ≥4/6 on the avoidability scale for either tier). The secondary outcome was the incidence of patient harm judged at least possibly avoidable for either tier (≥3/6).

### Descriptive analysis

Demographics of the prisoners (age, gender) and prisons (type and size) were summarised. The PatIent SAfety (PISA) classification system,^20^ which has an ontology aligned with the International Classification for Patient Safety,^26^ was used to deconstruct and code the written case narratives, record the incident outcome(s) and determine the degree(s) of avoidable harm (from low harm to significant harm (moderate harm, severe harm or death)). No inferences were made; only explicitly stated content in the case narratives was coded.

### Bias

To reduce reviewer bias, GP reviewers and the study team were blinded as to whether prisoners were in the enhanced sample or not. All reports were analysed by two academic GPs with patient safety experience (IJM and AC-S) once data collection was complete to agree on harm severity. Any narratives detailing ‘substandard care without resulting harm’ were reviewed again by IJM to ensure harms had not been missed, and areas of uncertainty were raised with the wider team.

### Expert case discussion

To contextualise avoidability ratings, IJM and AC-S discussed cases with experts from primary care (general practice, optometry, pharmacy, dentistry, nursing) and prisons (general practice, pharmacy, nursing and forensic psychiatry). These experts provided subject matter knowledge and awareness of clinical context to sense check, and to support community care (tier 1) and prison regime (tier 2) judgements, where required.

### Escalating concerns

When concerns about prisoner care were expressed by the GPs or study team, prison healthcare providers were notified to act as required. New concerns about ongoing care were raised, as opposed to examples of substandard care or harm that had already occurred.

Further information regarding the methodological approach is detailed within the published protocol,^19^ and there were no deviations. This study is reported in line with the REporting of studies Conducted using Observational Routinely collected health Data (RECORD) statement.

### Statistical analysis

Descriptive statistics were presented as frequencies and percentages. Percentages were expressed as valid percentages (i.e, if information was missing, it was excluded from the analysis). The incidence rate of avoidable harm was estimated (with 95% Poisson confidence intervals (CI)) using the frequency of avoidable harm as the numerator and patient-years as the denominator. Cases double-reviewed by GPs for avoidable harm were assessed for inter-rater reliability using the Cohen’s kappa statistic (with 95% CI), assessed between GPs, and the study team (determining the agreement of the presence of avoidable healthcare-associated harm).

We estimated the ‘minimum’ rate of healthcare-associated harm based on the number of avoidable harms within both the enhanced sample and the outsample reviewed by the GPs (numerator). Based on the observed number of harms from the outsample, we also estimated the ‘maximum’ incidence rate of harms if all cases had been reviewed, using the identified harms from the enhanced sample and the outsample, plus predicted harms from the remaining ‘unsampled’ prisoners (numerator). For both the ‘minimum’ and ‘maximum’ rates, the denominator was the total number of patient-years in the study. The sample of prisons examined was, although not a random sample, a broad representative of prisons nationally in terms of size, capacity and security. The minimum and maximum range of rates with 95% CIs were estimated to be representative of the entire national prison population.

Multilevel logistic regression models were fitted to identify prison-level characteristics that were associated with all harms experienced by prisoners within the enhanced sample during the census period.

Prison size (capacity) and security categories, age and deprivation level of the prison area were examined as characteristics associated with the frequency or nature of avoidable harm. To explore the variation of avoidable harm between and within prisons, we fitted two-level logistic regression models with prisoners (level 1) nested within prisons (level 2). Prisons were treated as random effects and the characteristics as fixed effects. An initial null model (model 1) was fitted with no fixed effects to estimate the variation explained between prisons, followed by a two-variable model (model 2) adjusted for age (centred at the median age) and deprivation (prisons in the most deprived quintile in England, based on the English Index of Multiple Deprivation).^27^A four-variable model (model 3) was then fitted adjusting for age and deprivation as well as prison capacity and prison category of security (ranked from A to D and female-only prisons, with A being the highest security and D the lowest). For all analyses, variance estimates of avoidable harm explained by each model, together with odds ratios (OR), 95% CIs and p values for each associated factor, were examined. P values were considered significant at the 5% level. Data were analysed using STATA V.16.1.^28^

## Results

### Descriptive statistics

Basic prison characteristics, which do not breach site anonymity, are shown in online supplemental table 2.

The nurse/doctor screeners screened 15 027 prisoner records. 6295 prisoner records met the inclusion criteria for the enhanced sample, with one record omitted from the review due to missing data, creating a sample of 6294 records, consisting of 5896 males, 396 females and two non-binary prisoners. Our ~10% outsample comprised 853 patients (832 males and 21 females). Within the 12-month census period, prisoners underwent multiple transfers, with one prisoner transferred twenty times between prisons, and another moved six times between the community and five times between prisons.

The total number of patient-years within the 12-month census period for all prisoners was 11 020 years (4883 years from the 6294 ‘enhanced’ prisoners, and 6137 years from the remaining ‘outsample’ 8732 prisoners).

**Figure 1:**
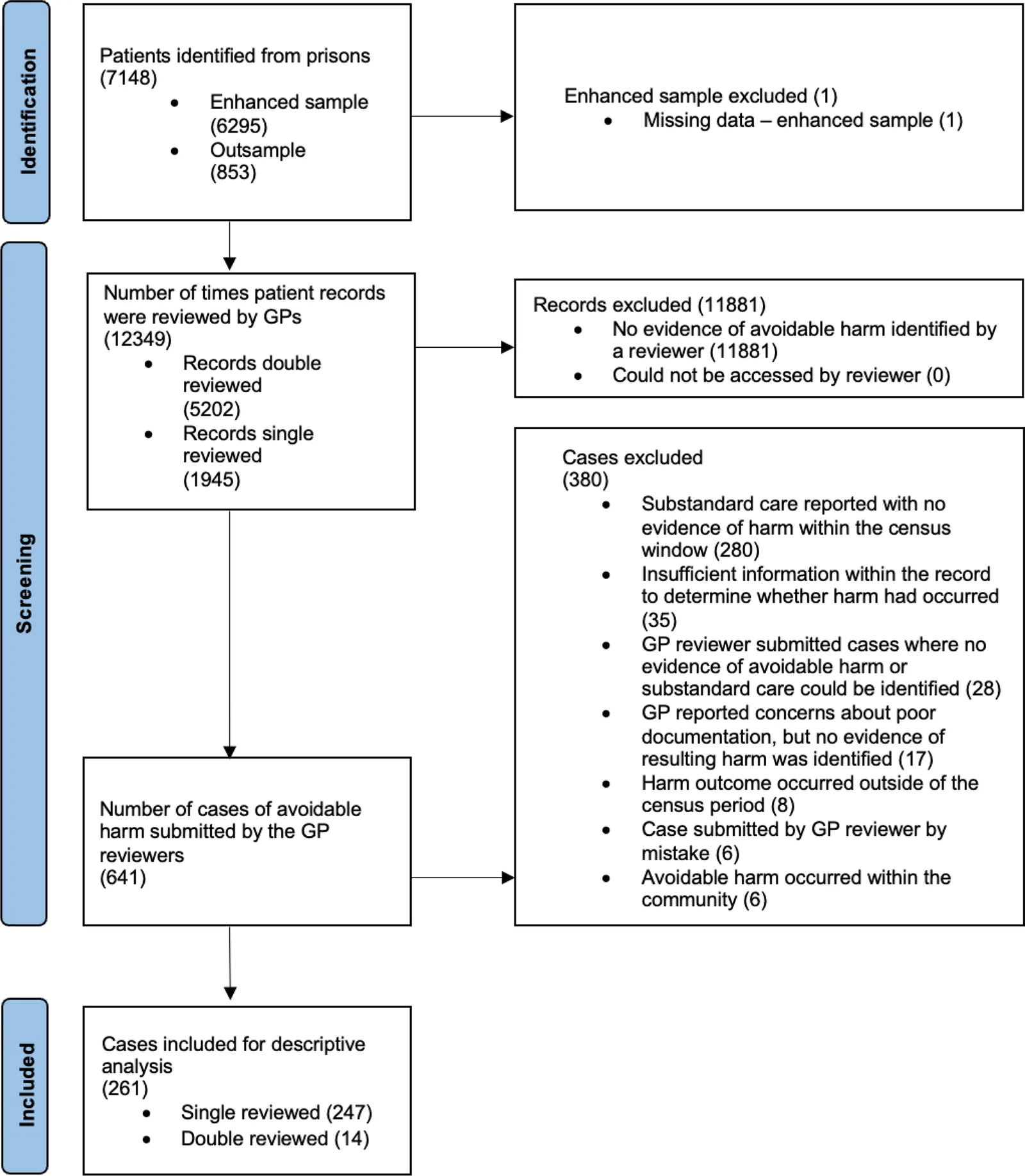
Flow chart to display case selection for analysis. GP, general practitioner.

Within the 7147 prisoner case notes (enhanced sample plus the outsample) reviewed by the GPs, there was evidence of healthcare-associated harm in 261 reports, with 14 cases submitted by both reviewers during double review (see figure 1), leading to 247 (of 7147, 3.5%) reports of harm after deduplication.

Most harms were derived from the enhanced sample (n=233, 94.3%) and a smaller proportion from the outsample (n=14, 5.7%), and were considered avoidable in nature. These harms were experienced by 244 prisoners, with one prisoner experiencing three safety incidents and another experiencing two. Of those, 234 were male (95.9%) and 10 were female (4.1%), aged between 20 and 88 (median age=42) years.

Of the 247 cases of avoidable harm, 211 were ‘significant’ harm (moderate, severe or death), 171 (69.2%) were probably avoidable, 204 were possibly avoidable (82.6%) and eight harms (0.1%) were definitely avoidable.

### Overall rate of healthcare-associated harm

Using the number of records reviewed by the GPs (all of the enhanced sample records and the 10% outsample records) and the 11 020 patient-years available for all prisoners screened, we estimated a ‘minimum’ incidence of all avoidable harms in prisons in England of 2241.4 (95% CI 1970.5 to 2539.0) per 100 000 patient-years (table 1), when assuming that no harm had occurred within prison records not reviewed.

**Table 1:** Number and incidence of avoidable harms overall and in subgroups

	n (% of 15 027 records)	Minimum* rate per 100 000 patient-years (95% CI)	Potential maximum† rate per 100 000 patient-years (95% CI)
All harms	247 (1.6)	2241.4 (1970.5 to 2539.0)	3412.0 (3075.8 to 3774.9)
Possibly avoidable harm (tier 1 or 2 judgement of 3+)	236 (1.6)	2141.6 (1877.0 to 2432.9)	3312.2 (2981.0 to 3670.0)
Probably avoidable harm (tier 1 or 2 judgement of 4+)	198 (1.3)	1796.7 (1555.2 to 2065.2)	2804.0 (2500.0 to 3134.7)
Definitely avoidable harm (tier 1 or 2 judgement of 6)	12 (0.08)	108.9 (56.3 to 190.2)	108.9 (56.3 to 190.2)
All ‘significant’ harms (death, severe, moderate)	211 (1.4)	1914.7 (1665.1 to 2191.2)	2922.0 (2611.5 to 3259.2)
Possibly avoidable ’significant’ harm (tier 1 or 2 judgement of 3+)	204 (1.4)	1851.2 (1605.9 to 2123.4)	2858.4 (2551.5 to 3192.2)
Probably avoidable ’significant’ harm (tier 1 or 2 judgement of 4+)	171 (1.1)	1551.7 (1327.9 to 1802.5)	2386.6 (2106.8 to 2693.1)
Definitely avoidable ’significant’ harm (tier 1 or 2 judgement of 6)	8 (0.05)	72.6 (31.3 to 143.0)	72.6 (31.3 to 143.0)
**Avoidable harm in the ‘enhanced’ sample**			
All harms	233 (1.6)	2114.3 (1851.5 to 2404.0)	3412.0 (3075.8 to 3774.9)
Possibly avoidable harm (tier 1 or 2 judgement of 3+)	222 (1.5)	2014.5 (1758.2 to 2297.7)	3312.2 (2981.0 to 3670.0)
Probably avoidable harm (tier 1 or 2 judgement of 4+)	186 (1.2)	1687.8 (1454.0 to 1948.6)	2804.0 (2500.0 to 3134.7)
Definitely avoidable harm (tier 1 or 2 judgement of 6)	12 (0.08)	108.9 (56.3 to 190.2)	108.9 (56.3 to 190.2)
All ‘significant’ harms (death, severe, moderate)	199 (1.3)	1805.8 (1563.6 to 2074.9)	2922.0 (2611.5 to 3259.2)
Possibly avoidable ’significant’ harm (tier 1 or 2 judgement of 3+)	192 (1.3)	1742.3 (1504.5 to 2006.9)	2858.4 (2551.5 to 3192.2)
Probably avoidable ’significant’ harm (tier 1 or 2 judgement of 4+)	161 (1.1)	1461 (1244.0 to 1704.9)	2386.6 (2106.8 to 2693.1)
Definitely avoidable ’significant’ harm (tier 1 or 2 judgement of 6)	8 (0.05)	72.6 (31.3 to 143.0)	72.6 (31.3 to 143.0)
**Community care (tier 1) judgement only ‘enhanced’ sample**			
Possibly avoidable harm (judgement of 3+)	195 (1.3)	1769.5 (1529.9 to 2036.1)	2885.7 (2577.2 to 3220.9)
Probably avoidable harm (judgement of 4+)	151 (1.0)	1370.2 (1160.4 to 1607.1)	2205.1 (1936.5 to 2500.5)
Definitely avoidable harm (judgement of 6)	8 (0.05)	72.6 (31.3 to 143.0)	72.6 (31.3 to 143.0)
Possibly avoidable ’significant’ harm (judgement of 3+)	169 (1.1)	1533.6 (1311.1 to 1783.0)	2368.4 (2089.8 to 2673.9)
Probably avoidable ’significant’ harm (judgement of 4+)	130 (0.9)	1179.7 (985.6 to 1400.8)	1923.8 (1673.5 to 2200.9)
Definitely avoidable ’significant’ harm (judgement of 6)	5 (0.03)	45.4 (14.7 to 105.9)	45.4 (14.7 to 105.9)
**Prison regime (tier 2) judgement only ‘enhanced’ sample**			
Possibly avoidable harm (judgement of 3+)	149 (1.0)	1352.1 (1143.7 to 1587.5)	2559.0 (2269.0 to 2875.8)
Probably avoidable harm (judgement of 4+)	81 (0.5)	735.0 (583.7 to 913.6)	1288.6 (1085.4 to 1518.8)
Definitely avoidable harm (judgement of 6)	6 (0.04)	54.5 (20.0 to 118.5)	54.5 (20.0 to 118.5)
Possibly avoidable ’significant’ harm (judgement of 3+)	131 (0.9)	1188.7 (993.9 to 1410.6)	2214.2 (1945.0 to 2510.1)
Probably avoidable ’significant’ harm (judgement of 4+)	72 (0.5)	653.4 (511.2 to 822.8)	1116.2 (927.6 to 1331.7)
Definitely avoidable ’significant’ harm (judgement of 6)	6 (0.04)	54.5 (20.0 to 118.5)	54.5 (20.0 to 118.5)
**Avoidable harm in the ’outsample’**			
All harms	14 (0.1)	127.0 (69.5 to 213.2)	1297.6 (1093.7 to 1528.6)
Possibly avoidable harm (tier 1 or 2 judgement of 3+)	14 (0.1%)	127.0 (69.5 to 213.2)	1297.6 (1093.7 to 1528.6)
Probably avoidable harm (tier 1 or 2 judgement of 4+)	12 (0.08)	108.9 (56.3 to 190.2)	1116.2 (927.6 to 1331.7)
Definitely avoidable harm (tier 1 or 2 judgement of 6)	0	–	–
All ‘significant’ harms (death, severe, moderate)	12 (0.08)	108.9 (56.3 to 190.2)	1116.2 (927.6 to 1331.7)
Possibly avoidable ’significant’ harm (tier 1 or 2 judgement of 3+)	12 (0.08)	108.9 (56.3 to 190.2)	1116.2 (927.6 to 1331.7)
Probably avoidable ’significant’ harm (tier 1 or 2 judgement of 4+)	10 (0.07)	90.8 (43.5 to 166.9)	925.6 (754.7 to 1123.6)
Definitely avoidable ’significant’ harm (tier 1 or 2 judgement of 6)	0	–	–

Possibly avoidable harm where judgement is 3+ on the avoidability scale, probably avoidable harm where judgement is 4+, definitely avoidable harm where judgement is 6.

* Minimum rate based on observed cases reviewed by GPs.

† Maximum rate if all study records were reviewed by GPs; rates are based on a denominator of 11 020 patient-years.

‡‘ Significant’ harms only (death, severe or moderate).

GP, general practitioner.

If the GPs had reviewed the remaining 7880 screened records, we estimated there could have been an additional 129 cases of avoidable healthcare-associated harm across the 18 prisons. Combined with the 14 cases of avoidable harm from the reviewed 10% outsample, this yielded an estimated total of 143 cases from the ‘unenhanced’ sample.

Therefore, the estimated incidence of avoidable harm could have reached a maximum rate of 3412.0 (95% CI 3075.8 to 3774.9) per 100 000 patient-years. Rates of harm by other subgroups, including probably avoidable and possibly avoidable harms, are presented in table 1.

The minimum rate of probably avoidable harm overall was estimated at 1796.7 (95% CI 1555.2 to 2065.2) per 100 000 patient-years and could be as high as 2804.0 (95% CI 2500.0 to 3134.7) per 100 000 patient-years when considering additional (unobserved) harms likely to have occurred among prisoners not included in our samples. The rate of possibly avoidable healthcare-associated harm was 2141.6 (95% CI 1877.0 to 2432.9) per 100 000 patient-years, and taking into account additional unobserved harms (as above) could have been up to 3312.2 (95% CI 2981.0 to 3670.0).

### Rate of avoidable harm by gender

Most cases of avoidable harm in the enhanced sample were experienced by men (n=223), resulting in a minimum rate of harm for males as 2097.0 (95% CI 1831.0 to 2319.1) per 100 000 patient-years. If all prisoner records had been reviewed, there could have been an additional 132 (14 from 10% outsample reviewed, plus 118 from the unsampled) cases of avoidable harm in men, with an estimated maximum rate of 3338.7 (95% CI 3000.4 to 3704.7).

Ten cases of avoidable harm were experienced by women, giving a minimum rate of 2604.2 (95% CI 1248.8 to 4789.2) per 100 000 patient-years. We did not identify additional cases of avoidable harm for women in the outsample; therefore, no adjustment was made to estimate a maximum rate of harm. Further avoidability ratings by patient gender and age are included in online supplemental tables 4 and 5.

The two non-binary prisoners did not experience any avoidable harms.

### Inter-rater reliability

There was 92.9% agreement between GP reviewers regarding the presence of avoidable harm, comparing 3447 cases submitted by GPs within the enhanced sample, with a Cohen’s kappa (0.12, 95% CI 0.06 to 0.17) indicating slight agreement. Furthermore, there was 69.0% agreement in 335 cases between GP reviewers and the study team regarding the presence of avoidable harm within the enhanced sample cases, Cohen’s kappa (0.37, 95% CI 0.29 to 0.46), indicating fair agreement, with GPs often proposing harm, and the study team considering the narratives to contain substandard care without resulting harm.

### Incident outcome and harm severity

Two-thirds of the patient safety incidents contained more than one outcome (162 of 247 cases, 66.0%), with 442 outcomes documented in total. Two-fifths of reported outcomes involved prisoners experiencing discomfort and pain (99 of 247, 40.1%) and delays in management, assessment or treatment (91, 37.0%).

Deterioration of medical conditions was also frequently reported (76, 30.8%), and this involved worsening respiratory conditions, including COVID-19-related pneumonia (12 of 76, 15.8%) and asthma (5, 16.0%).

Anonymised examples for each type of harm severity can be found in table 2.

**Table 2:** Harm severity and patient examples

Harm severity *As defined by NHS England*^24^	Number of harms (%) of safety incidents	Patient vignette
Death: *Death that is directly attributed to the patient safety incident*	27 (10.9)	*“*On arrival to the prison, the prisoner stated that they would hang themselves, but no Assessment, Care in Custody and Teamwork plan* (ACCT) was started, and they were not referred to the mental health team for support. After [period of time] they were moved to the segregation unit, and when unobserved hanged themselves within the cell*.”*
Severe harm: *Harm that is permanent or long term*	27 (10.9)	*“*Advanced cancer was missed for a patient who had presented to the healthcare team several times, with change in bowel habit, abdominal pain and feeling unwell. Healthcare professionals thought this was related to drug-seeking behaviour and did not appropriately examine or refer the patient. When the patient collapsed, and was transferred to the hospital via ambulance, they needed emergency surgery and a prolonged stay in the intensive care unit.*”*
Moderate harm: *Harm that is considered short term in nature: patient(s) required further treatment or procedure*	157 (63.6)	*“*Following an injury, the GP was concerned that there was a fracture in [limb]. Despite advising that the patient needed to be transferred immediately to the [Emergency Department (ED)] for an X-ray, the patient did not attend for nearly two weeks, despite a lot of pain. When they were seen in ED, a fracture was confirmed, a cast was applied, and they were followed up in fracture clinic.”
Mild harm: *Harm that has a short-term impact on physical, mental or social functioning, expected to resolve in a few hours*	18 (7.3)	*“*A prisoner, with a previous history of impulsive medication overdose and self-harm, was given a supply of analgesia (in-possession). No medication review took place for several months, and they stockpiled hundreds of tablets, taking an unknown number as an overdose and were admitted to ED for an assessment. Following blood tests, fluids and observations, they were discharged that day, and all in-possession medications were subsequently stopped.*”*
Emotional harm (short term): *Experienced transient emotional distress but no long-term consequences*	12 (4.9)	*“*A prisoner sought support for their sleep and anxiety, leading up to their court appearance. Despite healthcare professionals (HCPs) advising that they would share sleep hygiene advice, support and relaxation activities, this did not happen, causing the prisoner to state they felt very upset and not listened to. Following their court appearance, they were seen by a HCP again, and stated that their anxiety and their insomnia had ceased*.”*
Low harm: *Was harmed but required no or minimal intervention*	2 (0.8)	*“*A patient with a pressure ulcer was seen by the tissue viability team who advised that a specific dressing should be used. At their next appointment, they realised that the wrong dressing had been applied, and so a new dressing was applied which helped it to heal quicker.*”*
Unclear: *Unclear severity, despite harm*	4 (1.6)	*“*A prisoner was seen by a prison GP, with dysphagia [difficulty swallowing], abdominal pain and weight loss. They were referred to the appropriate speciality, via the urgent suspected cancer pathway, but when they arrived at the hospital, the investigations were abandoned due to the patient being double-cuffed. No follow up appointment was received from secondary care, and this was not chased by the prison healthcare team for several months, until after the patient had re-presented with further weight loss and concerns. The prisoner was released from prison before any investigations took place*.”*

*ACCT: plan to support vulnerable people within prison, that is, if at risk of self-harm or suicide.

ACCT, Assessment, Care in Custody and Teamwork; ED, Emergency Department; GP, general practitioner; HCP, healthcare professional; NHS, National Health Service.

Most cases of healthcare-associated harm resulted in moderate harm (157 of 247 cases, 63.6%), followed by severe harm (27, 10.9%) and death (27, 10.9%).

The most frequent outcomes were associated with the highest rating of harm severity, that is, discomfort and pain (99/247, 40.1%) were associated with moderate harm (84 of 99, 84.8%), severe harm (10, 10.1%) and incidents leading to death (1, 1.0%).

The second most frequent outcome was delays in management, assessment or treatment, which were also associated with moderate harm most frequently (64 of 91, 70.3%), followed by severe harm (12, 13.1%) and death (8, 8.8%). Cases involving delays in management, assessment or treatment also included emotional harm (7, 7.7%), with no instances of low or mild harm.

A comparison of incident outcomes by harm severity is included in online supplemental table 6.

### Avoidability

All 247 cases contained evidence of avoidability (with community care (tier 1) or prison regime (tier 2) judgement ratings of ≥2/6). 198 (80.2%) cases were probably avoidable (community care or prison regime rating ≥4/6), 14 (5.7%) were definitely avoidable (community care or prison regime rating ≥6/6) and 236 (95.5%) were possibly avoidable (community or prison regime judgement rating ≥3/6).

Vignettes (based on case amalgamation) highlighting rating extremes are included in table 3.

**Table 3:** Vignettes of community and prison care comparisons using avoidability ratings

Avoidability judgement		Description	Example case
Community care (tier 1)	1/6	Totally unavoidable from a community care perspective, but totally avoidable from a prison regime perspective	*“*After being transferred to hospital from prison following a significant chest infection, the patient was double-cuffed within the bed, which restricted the healthcare professional’s ability to support the patient and ensure comfort and dignity; they were unable to be fully washed and turned appropriately for skin care. The healthcare team raised their concerns about this and asked for consideration of single cuffing / enhanced security to allow for their care needs, but this was declined by the officers. The prison refused any alternatives when this was escalated by the Medical Director to the prison Governor. The patient’s skin broke down around the cuffs and they developed pressure sores. After [time], the patient developed a pulmonary embolism and died*.”*
Prison regime (tier 2)	6/6		
Community care (tier 1)	6/6	Totally unavoidable from a prison regime perspective, but totally avoidable from a community care perspective	*“*On admission to prison, a patient was offered hepatitis screening, and found to be positive for hepatitis C. Despite the healthcare team receiving the result and being aware of the diagnosis, the healthcare professional stated that a task would be sent to refer the patient appropriately to Hepatology, and a care plan would be required. However, these tasks were never sent. The patient was released from prison some weeks later, and, after returning to the same prison, the team recognised that they had hepatitis C but had not received any treatment. No referral was sent, but blood tests were repeated. Despite significantly abnormal liver function tests and active hepatitis serology, the patient was not reviewed again, and a referral was still not sent. Only when the patient was seen for a different condition, were the bloods re-reviewed, and an appropriate referral and treatment arranged.*”*
Prison regime (tier 2)	1/6		

Details regarding the multilevel regression model are included in online supplemental tables 7 and 8.

## Discussion

### Principal findings

The estimated ‘maximum’ incidence of all avoidable harms in prisons in England was 3412.0 (95% CI 3075.8 to 3774.9) per 100 000 patient-years. Extrapolating these findings to the total prison population, which in 2024 was ~98 000,^29^ there are likely ~3000–3700 cases of avoidable healthcare-associated harm annually.

The most frequently reported types of patient safety outcomes involved prisoners experiencing discomfort and pain, followed by delays in management, assessment or treatment. Most cases resulted in significant patient harm, which consisted of moderate harm, severe harm or death.

### Strengths and weaknesses of the study

This study is, to our knowledge, the first to examine avoidable patient harm within the prison healthcare system through case note review. The nurse, doctor and GP reviewers were experienced researchers from previous studies^23 30 31^ and had significant training to complete the screening/case note reviews, with high-quality reporting.

In keeping with our former study in primary care,^23^ there was agreement between GP reviewers, but not necessarily accuracy of detecting harm, reflected in the low kappa coefficients. The subjectivity of case note reviews is well known,^32^ with a systematic review focused on kappa coefficients highlighting concerns about publication bias and inter-rater concordance, with lower kappa values found with implicit case note reviews such as ours.^33^

To ensure high sensitivity of cases identified (at the cost of accepting a lower level of specificity), all narratives (including substandard care) submitted by the GPs were reviewed by the study team. However, due to the practical/financial restrictions of an open case note review, the GPs could not review all of the patient records, and the GPs did not submit any narratives which they considered that a patient safety incident had not occurred. Overall, it is highly likely that our cases of harm, and therefore the true rate of avoidable harm, may be even higher, and the scale of the problem could be worse. We have therefore provided ‘minimum’ and ‘maximum’ rates of harms based on records the GPs had not reviewed in our estimates, to indicate at least how high these rates could be.

There was also a risk of reporting bias by the GP reviewers,^32^ as they were aware that the study aimed to estimate the incidence of avoidable healthcare-associated harm, in keeping with our hypotheses associated with the enhanced criteria, and that our previous primary care study found no harms within the outsample.^23^ To address this, reviewers and researchers were blinded from screening outcomes.

Reviewers advised that medical records were often incomplete, correspondence between healthcare teams was frequently absent and many of the medical entries were sparse or of poor quality. This has also been seen in other studies that focused on healthcare quality in prisons,^34–36^ and it is possible that care was given and not documented.

### Comparison with primary care

Our prior study analysing healthcare-associated harm in primary care in England^23 30 31^ estimated that the rate of probably avoidable significant harm was 35.6 per 100 000 patient-years. Comparing this study to match Avery *et al (2021)*^23^ using their enhanced sample only at a rate of 1461 (95% CI 1244 to 1705) per 100 000 patient-years, or a rate of 2387 (95% CI 2106 to 2693) per 100 000 patient-years if all records had been reviewed by GPs, it could be interpreted that prisoners are at a 41–67 times greater risk of experiencing probably avoidable significant healthcare-associated harm, compared with care delivered in the community.

However, these two studies should not be directly compared without caution, as the populations were different, and they used different screening methods. Our prison study did not exclusively include ‘significant’ diagnoses (e.g. stroke), and we included all harms, arising from all diseases, at different degrees of maturation, focusing on what was probably or possibly avoidable.

### Recommendations for future research

This large-scale case note review of 18 prisons in England has highlighted that prisoners are at risk of avoidable healthcare-associated harm. Our study has additionally focused on the incident types, contributory factors and the sensitivities of the enhanced criteria, and has gained input from prisoners and staff within its final study phase, interviewing prisoners and prison staff (findings to be published elsewhere).

This study promotes the continued value of judging care in secure environments against what should be received within the community, and considering how and where the prison regime impacts care that can be delivered to improve standards in prisons. This would allow prisons to explore the challenges and consider safe and effective care. It would also allow healthcare teams to better work with prisoners, prison teams and more effectively within prison systems, so that patient safety is a priority.

### Implications for policy and healthcare teams

Using our findings about the avoidable healthcare-associated harm in prisons, particularly concerning prisoners’ physical and mental health, focus is needed to address the systemic failures contributing to such harmful outcomes, especially for the management of long-term conditions, and supporting prisoners at risk of self-harm and suicide.

Given the high turnover and regular transfer of prisoners, and concerns regarding the poor medical documentation, administrative support, training and time are needed to ensure full utilisation of electronic medical records. This might include coding and recall notices to support healthcare delivery and monitoring of long-term conditions. Standard operating procedures may also support medical tasks and referrals to be completed in a timely manner, and results communicated to patients.^37 38^

The notion of equivalence must be operationalised. Beyond research studies, regular case note review, using tools in this study, should support improvement, driven by regular, structured appraisal of care delivery. We focused on avoidable harm to highlight opportunities for improving patient safety through targeted actions. While decisions surrounding avoidability can change as healthcare advances, this approach encourages ongoing innovation and continuous improvement in reducing harm.^39^

Learning should inform staff training, continuous professional development, audit and improvement. Buy-in is needed into the mindset that achieving equitable health outcomes will have greater societal benefits, with a need to address tensions between security and healthcare within secure environments. Ultimately, this will reduce strain on the NHS by reducing emergency department attendance, operationalising efforts of prevention, as opposed to cure.

## Conclusion

This study has highlighted that prisoners are at risk of avoidable healthcare-associated harm, and substantive improvement in prison healthcare delivery is needed to ensure patient safety. Efforts in prisons will not give prisoners ‘special treatment’, not afforded to the rest of the population, but will reduce health inequalities faced by a population with complex healthcare needs.

Our findings highlight the need for continued evaluation of care within prisons against what is received in the community, and consideration of how and where the prison regime affects care delivery, to improve healthcare standards. This would allow prisons to consider the challenges and how care can be improved to become safe. It will also allow healthcare teams to better work with prison colleagues and more effectively within prison systems, so that patient safety in prisons is a priority.

## Supplementary material

10.1136/bmjqs-2025-019935 online supplemental file 1

10.1136/bmjqs-2025-019935 online supplemental file 2

## Data Availability

No data are available.
