# Supplementary material for: The incidence of avoidable healthcare-associated harm in prisons in England: a retrospective case note review
Source: BMJ Qual Saf. 2026 May 7;35(9):e019935. doi: 10.1136/bmjqs-2025-019935 (PMC13539849; doi:10.1136/bmjqs-2025-019935)
Supplement: Supplementary file 1 [file bmjqs-35-9-s001.pdf]

Supplementary Table 1. Avoidability scale: Adapted from Keers et al. (11)

Rating	Category	Description
1	Definitely unavoidable	Virtually no evidence of avoidability
2	Unavoidable	Slight to moderate evidence of avoidability
3	Possibly avoidable	Possibly avoidable, less than 50-50 but close call
4	Probably avoidable	Probably avoidable, more than 50-50 but close call
5	Avoidable	Strong evidence of avoidability
6	Definitely avoidable	Virtually certain evidence of avoidability

Supplementary Table 2. Prison category and census day

Prison Identifier	Prison category	Census day	Primary healthcare provider
A	B	19/07/21	Private Provider
B	B	03/05/21	Private Provider
C	Female (closed)	10/05/21	Private Provider
D	A	22/03/21	Not for profit social business
E	B	12/07/21	Private Provider
F	D	22/02/21	Not for profit social business
G	B	03/05/21	Private Provider
H	B	22/02/21	Not for profit social business
I	A	08/03/21	NHS Trust
J	C	14/06/21	Private Provider
K	C	24/05/21	Private Provider
L	C	26/11/20	NHS Trust
M	Female (closed)	22/02/21	Not for profit social business
N	D	26/11/20	NHS Trust
O	A	24/06/21	Private Provider
P	B	24/05/21	NHS Trust
Q	C	24/05/21	NHS Trust
R	B	12/07/21	Private Provider

Category A: high security prisons housing male prisoners who, if they were to escape, pose the most threat to the public, the police or national security.
Category B: local or training prisons. Local prisons house prisoners directly from court in the local area (sentenced or on remand), and training prisons hold long-term and high-security prisoners.
Category C: training and resettlement prisons; most prisoners are in a category C. They provide the opportunity to develop skills to aid finding work and resettlement back into the community.
Category D: open prisons with minimal security. Eligible prisoners spend most of their day away from the prison on licence to carry out work, education or for other resettlement purposes. Open prisons only house prisoners that have been risk-assessed and deemed suitable for open conditions.
Women: held in either closed or open conditions, according to their risks and needs. Those considered high risk are categorised as ‘restricted status’, meaning they can only be held in a closed prison.

Supplementary Table 3. Enhanced sample criteria

• A new significant health problem as defined in our primary care study(15).
• Multimorbidity.
• Allergies or adverse drug reactions.
• Polypharmacy (>10 regular medications).
• Admission to an Emergency Department or an unscheduled admission to hospital / a mental health facility.
• Acute health problem following substance use.
• Died during the study period.
• Did not attend scheduled hospital appointments.
• Required attention from an out of hours healthcare professional or paramedic.
• Abnormalities in the following blood results:<br>○ Urea and electrolytes: eGFR change of ≥5 in patients with an eGFR <45 (i.e., chronic kidney disease stage 3b).<br>○ Aspartate aminotransferase or alanine aminotransferase >150.<br>○ Haemoglobin <100 g/L.

Supplementary Table 4. Number and incidence of avoidable harm by gender*

	Male prisoners		Female prisoners	
	N (%)	Rate per 100,000 patient-years (95% CI)	N (%)	Rate per 100,000 patient-years (95% CI)
Total study sample	13,771		606	
Enhanced sample	5,897		395	
Number of patient years	10,633		384	
All harms in the enhanced sample	223 (1.6%)	2097 (1831 - 2391)	10 (1.7%)	2604 (1249 - 4789)
Possibly avoidable harm (Tier 1 or 2 judgement of 3+)	213 (1.5%)	2003 (1743 - 2291)	9 (1.5%)	2344 (1072 - 4449)
Probably avoidable harm (Tier 1 or 2 judgement of 4+)	177 (1.3%)	1665 (1428 - 1929)	9 (1.5%)	2344 (1072 - 4449)
Definitely avoidable harm (Tier 1 or 2 judgement of 6)	12 (0.1%)	113 (58 - 197)	0	-
All 'significant' harms in the enhanced sample	192 (1.4%)	1806 (1559 - 2080)	7 (1.2%)	1823 (733 - 3756)
Possibly avoidable 'significant' harm (Tier 1 or 2 judgement of 3+)	186 (1.4%)	1749 (1507 - 2020)	6 (1.0%)	1563 (573 - 3401)
Probably avoidable 'significant' harm (Tier 1 or 2 judgement of 4+)	161 (1.1%)	1458 (1237 - 1706)	6 (1.0%)	1563 (573 - 3401)
Definitely avoidable 'significant' harm (Tier 1 or 2 judgement of 6)	8 (0.1%)	75 (32 - 148)	0	-
**Community care (Tier 1) judgement only**				
Possibly avoidable harm (judgement of 3+)	186 (1.4%)	1749 (1507 - 2020)	9 (1.5%)	2344 (1072 - 4449)
Probably avoidable harm (judgement of 4+)	142 (1.0%)	1335 (1125 -1574)	9 (1.5%)	2344 (1072 - 4449)
Definitely avoidable harm (judgement of 6)	8 (0.1%)	75 (32 - 148)	0	-
Possibly avoidable 'significant' harm (judgement of 3+)	163 (1.2%)	1533 (1307 - 1787)	6 (1.0%)	1563 (573 - 3401)
Probably avoidable 'significant' harm (judgement of 4+)	124 (0.9%)	1166 (970 - 1390)	6 (1.0%)	1563 (573 - 3401)
Definitely avoidable 'significant' harm (judgement of 6)	5 (0.4%)	47 (15 - 110)	0	-
**Prison regime (Tier 2) judgement only**				

Possibly avoidable harm (judgement of 3+)	141 (1.0%)	1326 (1116 - 1564)	8 (1.3%)	2083 (899 - 4105)
Probably avoidable harm (judgement of 4+)	79 (0.6%)	743 (588 - 926)	2 (0.3%)	521 (63 - 1881)
Definitely avoidable harm (judgement of 6)	6 (<0.1%)	56 (21 - 123)	0	-
Possibly avoidable 'significant' harm (judgement of 3+)	131 (0.9%)	1176 (979 - 1401)	6 (1.0%)	1563 (573 - 3401)
Probably avoidable 'significant' harm (judgement of 4+)	70 (0.5%)	658 (513 - 832)	2 (0.3%)	521 (63 - 1881)
Definitely avoidable 'significant' harm (judgement of 6)	6 (<0.1%)	56 (21 - 123)	0	-

possibly harm where judgement is 3+ on the avoidability scale, probably harm where judgement is 4+, definitely harm where judgement is 6, ^'significant' harms only (death, severe or moderate), rates are based on patient years as denominator, CI = confidence interval
*The two non-binary prisoners did not experience any avoidable harms and are therefore not included within this table.

Supplementary Table 5. Number and incidence of avoidable harm by age groups

	Under 25 years old		25-44 years		45-64 years		65 years or over	
	N (%)	Rate per 100,000 patient-years (95% CI)	N (%)	Rate per 100,000 patient-years (95% CI)	N (%)	Rate per 100,000 patient-years (95% CI)	N (%)	Rate per 100,000 patient-years (95% CI)
Total study sample	1,799		9,318		3,215		676	
Enhanced sample	531 (30%)		3,652 (39%)		1,628 (51%)		476 (70%)	
Number of patient years	1,126		6,744		2,548		586	
All harms in the enhanced sample	16 (0.9%)	1421 (812 - 2308)	119 (1.3%)	1765 (1462 - 2112)	78 (2.4%)	3061 (2420 - 3821)	20 (3.0%)	3413 (2085 - 5271)
Possibly avoidable harm (Tier 1 or 2 judgement of 3+)	16 (0.9%)	1421 (812 - 2308)	112 (1.2%)	1661 (1367 - 1998)	74 (2.3%)	2904 (2280 - 3646)	20 (3.0%)	3413 (2085 - 5271)
Probably avoidable harm (Tier 1 or 2 judgement of 4+)	15 (0.8%)	1332 (746 - 2197)	93 (1.0%)	1379 (1113 - 1689)	61 (1.9%)	2394 (1831 - 3075)	17 (2.5%)	2901 (1690 - 4645)
Definitely avoidable harm (Tier 1 or 2 judgement of 6)	1 (0.1%)	89 (2 - 495)	7 (0.1%)	104 (42 - 214)	2 (0.1%)	78 (10 - 284)	2 (0.3%)	341 (41 - 1233)
All 'significant' harms in the enhanced sample	12 (0.7%)	1066 (551 - 1862)	104 (1.1%)	1542 (1260 - 1869)	66 (2.1%)	2590 (2003 - 3295)	17 (2.5%)	2901 (1690 - 4645)
Possibly avoidable 'significant' harm (Tier 1 or 2 judgement of 3+)	12 (0.7%)	1066 (551 - 1862)	99 (1.1%)	1468 (1193 - 1787)	64 (2.0%)	2512 (1934 - 3207)	17 (2.5%)	2901 (1690 - 4645)
Probably avoidable 'significant' harm (Tier 1 or 2 judgement of 4+)	11 (0.6%)	977 (488 - 1748)	82 (0.9%)	1216 (967 - 1509)	53 (1.6%)	2080 (1558 - 2721)	15 (2.2%)	2560 (1433 - 4222)
Definitely avoidable 'significant' harm (Tier 1 or 2 judgement of 6)	0 (0%)	-	5 (0.1%)	74 (24 - 173)	1 (<0.1%)	39 (1 - 219)	2 (0.3%)	341 (41 - 1233)
**Community care (Tier 1) judgement only**								
Possibly avoidable harm (judgement of 3+)	16 (0.9%)	1421 (812 - 2308)	97 (1.0%)	1438 (1166 - 1754)	66 (2.1%)	2590 (2003 - 3295)	16 (2.4%)	2730 (1561 - 4434)
Probably avoidable harm (judgement of 4+)	10 (0.6%)	888 (426 - 1633)	74 (0.8%)	1097 (862 - 1378)	54 (1.7%)	2119 (1592 - 2765)	13 (1.9%)	2218 (1181 - 3794)
Definitely avoidable harm (judgement of 6)	0	-	6 (0.1%)	89 (33 - 194)	2 (0.1%)	78 (10 - 284)	0	-
Possibly avoidable 'significant' harm (judgement of 3+)	12 (0.7%)	1066 (551 - 1862)	85 (0.9%)	1260 (1007 - 1558)	58 (1.8%)	2276 (1728 - 2943)	14 (2.1%)	2389 (1306 - 4008)
Probably avoidable 'significant' harm (judgement of 4+)	7 (0.4%)	622 (250 - 1281)	64 (0.7%)	949 (731 - 1212)	47 (1.5%)	1845 (1355 - 2453)	12 (1.8%)	2048 (1058 - 3577)
Definitely avoidable 'significant' harm (judgement of 6)	0 (0%)	-	4 (<0.1%)	59 (16 - 152)	1 (<0.1%)	39 (1 - 219)	0 (0%)	-
**Prison regime (Tier 2) judgement only**								
Possibly avoidable harm (judgement of 3+)	12 (0.7%)	1066 (551 - 1862)	77 (0.8%)	1142 (901 - 1427)	46 (1.4%)	1805 (1322 - 2408)	14 (2.1%)	2389 (1306 - 4008)

Probably avoidable harm (judgement of 4+)	8 (0.4%)	710 (307 - 1400)	47 (0.5%)	697 (512 - 927)	19 (0.6%)	746 (449 - 1164)	7 (1.0%)	1195 (480 - 2461)
Definitely avoidable harm (judgement of 6)	1 (0.1%)	89 (2 - 495)	2 (<0.1%)	30 (4-107)	1 (<0.1%)	39 (1-219)	2 (0.3%)	341 (41 - 1233)
Possibly avoidable 'significant' harm (judgement of 3+)	8 (0.4%)	710 (307 - 1400)	71 (0.8%)	1053 (822 - 1328)	39 (1.2%)	1531 (1088 - 2092)	13 (1.9%)	2218 (1181 - 3794)
Probably avoidable 'significant' harm (judgement of 4+)	6 (0.3%)	533 (196 - 1160)	43 (0.5%)	638 (461 - 859)	17 (0.5%)	667 (389 - 1068)	6 (0.9%)	1024 (376 - 2229)
Definitely avoidable 'significant' harm (judgement of 6)	1 (0.1%)	89 (2 - 495)	2 (<0.1%)	30 (4-107)	1 (<0.1%)	39 (1-219)	2 (0.3%)	341 (41 - 1233)

possibly avoidable harm where judgement is 3+ on the avoidability scale, probably where judgement is 4+, definitely where judgement is 6, ^'significant' harms only (death, severe or moderate), rates are based on age-specfic patient years as denominator, CI = confidence interval

Supplementary Table 6. Comparison of incident outcome by harm severity.

		Overall harm severity (247 incidents)						
		Low harm<br>2 (0.8)	Unclear<br>4 (1.6)	Emotional harm<br>12 (4.9)	Mild harm<br>18 (7.3)	Moderate harm<br>157 (63.6)	Severe harm<br>27 (10.9)	Death<br>27 (10.9)
**Outcome type (up to four coded per incident)**	**N (%)**							
Discomfort / pain	99 (40.0)		1	1	2	84	10	1
Delays in management, assessment or treatment	91 (36.8)			7		64	12	8
General deterioration / progression of condition	76 (30.8)		1		5	48	13	9
Death	25 (10.1)							25
Elective surgical procedure	23 (9.3)			1		16	6	
Hospital admission	21 (8.5)					10	7	4
Psychological difficulty	15 (6.1)			2	1	11	1	
Repeated tests /procedure / additional treatment	15 (6.1)		1		5	6	3	
Anxiety	10 (4.0)			7	2	1		
Poor diabetic control	7 (2.8)				2	5		
Self-harm	6 (2.4)				2	2		2
Upset / emotional distress	4 (1.6)			4				
Requiring specialist treatment	4 (1.6)					2	2	
Seizure(s)	4 (1.6)					4		
Infection	3 (1.2)				1	1	1	

Admission to ITU/HDU	3 (1.2)						2	1
Emergency surgery is required	3 (1.2)						3	
Complaint	3 (1.2)			1	1	1		
Changes in physiological parameters	2 (0.8)					2		
Other wound / ulcer	2 (0.8)	1				1		
Diabetic ulcer	2 (0.8)					2		
Bleeding	2 (0.8)		1				1	
Medication withdrawal symptoms	2 (0.8)					2		
Nausea / vomiting	2 (0.8)				2			
Suicide	2 (0.8)							2
Wrong treatment	2 (0.8)	1				1		
Unclear outcome	1 (0.4)		1					
Thrombosis	1 (0.4)					1		
Pulmonary Embolism	1 (0.4)					1		
Stroke	1 (0.4)					1		
DKA – Diabetic ketoacidosis	1 (0.4)						1	
Constipation	1 (0.4)					1		
Long term neurological condition	1 (0.4)					1		
Treatment changed	1 (0.4)				1			
Missed doses of medication	1 (0.4)							1
Medication overdose	1 (0.4)				1			
Delays in using facilities	1 (0.4)				1			
Progression of eye condition	1 (0.4)					1		
Transfer of patient to alternative location	1 (0.4)					1		
Uncontrolled unspecified physical symptoms	1 (0.4)					1		

Appendix 7. Multi-level regression model

Following analysis of rates of harm within each prison (see Supplementary Table 7), we firstly fitted the null model (model 1) with prisons as a random effect and no fixed effects, giving a prison-level variance of 0.656 (Table 4). When adding age and deprivation to the model (model 2), the estimated variance was 0.600, with a reduction of prison-level variability of 8.5% compared with the null model. With the subsequent addition of two further variables relating to prison population size (capacity) and the prison category of security, the estimated prison-level variance was 0.311 (model 3), indicating a further reduction in the prison level variance from model 2 to model 3 of 48%.

This indicated that the presence or absence of avoidable harm varied not only between prisons, but also between prison capacity and security category. However, the factors included in the multi-level logistic regression analysis showed that only age was a significant predictor of avoidable harm, with increasing likelihood of avoidable harm with increasing age of patients (OR = 1.017, 95% CI: 1.008 - 1.027, $p < 0.001$) (Supplementary Table 7). No other variables were statistically significant at the 5% level. However, there may be other unmeasured factors associated with harm, and we aim to examine some of these factors in future work.

Within our multi-level analysis, we examined deprivation levels not of the prisoners but of the prison itself, whose prison staff are likely to reside in the area. It was the best available deprivation metric and though it was not significant we would exercise caution in its interpretation, considering whether differences in rates of harm between prisons were associated with areas of deprivation.

Supplementary Table 7: Results of multi-level logistic regression analysis

Predictor	Number in the enhanced sample	Number with at least one avoidable harm (%)	Coefficient	Odds ratio (OR)	95% CI	P-value	Prison level variance
			**Model 1: Null model**				**0.6559**
**Constant**	6294	230 (4%)	-3.432			<0.001	
			**Model 2: Two-variable model**				**0.5999**
**Age (centred)***			0.017	1.018	1.008 - 1.027	<0.001	
**Prison deprivation^**	1756	54 (3%)	-0.320	0.726	0.301 - 1.754	0.477	
			**Model 3: Four-variable model**				**0.3117**
**Age (centred)***			0.017	1.017	1.008 - 1.027	<0.001	
**Prison deprivation^**	1756	54 (3%)	-0.246	0.782	0.288 - 2.124	0.630	
**Prison capacity**							
Small (<650)	1001	65 (6%)		*Baseline*			
Medium (650-1000)	2108	73 (3%)	-1.212	0.298	0.046 - 1.937	0.205	
Large (over 1000)	2955	93 (3%)	-1.509	0.221	0.026 - 1.907	0.170	
**Prison category**							
A	678	41 (6%)		*Baseline*			
B	2450	91 (4%)	0.600	1.822	0.373 - 8.892	0.458	
C	2273	67 (3%)	-0.055	0.947	0.142 - 6.298	0.955	
D	282	21 (7%)	-0.394	0.674	0.185 - 2.455	0.550	
Female only	381	10 (3%)	-1.230	0.292	0.077 - 1.106	0.070	
**Age centred at 38 yrs; ^represents the % in the most deprived quintile*							

Supplementary Table 8. Frequency of avoidable harms by prison

Prison	Enhanced sample	Outsample	All harms	All 'Possibly' avoidable harms	Avoidable harms (enhanced sample)	Avoidable harms (outsample)	At least one avoidable harm	At least one avoidable harm (enhanced sample)
A	448	70	19	19	19	0	19	19
B	384	75	15	14	15	0	15	15
C	157	7	3	1	3	0	3	3
D	170	39	19	13	17	2	19	17
E	319	26	13	8	13	0	13	13
F	159	30	1	1	1	0	1	1
G	278	84	13	9	10	3	13	10
H	374	40	17	16	17	0	17	17
I	347	28	8	5	7	1	8	7
J	365	46	9	7	9	0	9	9
K	1077	103	48	33	46	2	48	46
L	498	55	4	2	2	2	4	2
M	234	14	7	5	7	0	7	7
N	144	12	20	20	20	0	20	20
O	202	42	19	15	17	2	19	17
P	375	42	19	17	19	0	16	16
Q	400	59	12	6	10	2	12	10
R	363	81	1	1	1	0	1	1
**Total**	**6,294**	**853**	**247**	**192**	**233**	**14**	**244**	**230**
