# Supplementary material for: The incidence of avoidable healthcare-associated harm in prisons in England: a retrospective case note review
Source: BMJ Qual Saf. 2026 May 7;35(9):e019935. doi: 10.1136/bmjqs-2025-019935 (PMC13539849; doi:10.1136/bmjqs-2025-019935)
Supplement: Supplementary file 2 [file bmjqs-35-9-s002.pdf]

The RECORD statement – checklist of items, extended from the STROBE statement, that should be reported in observational studies using routinely collected health data.

	Item No.	STROBE items	Location in manuscript where items are reported	RECORD items	Location in manuscript where items are reported
**Title and abstract**					
	1	(a) Indicate the study's design with a commonly used term in the title or the abstract (b) Provide in the abstract an informative and balanced summary of what was done and what was found	Page 1: Title page	RECORD 1.1: The type of data used should be specified in the title or abstract. When possible, the name of the databases used should be included.<br><br>RECORD 1.2: If applicable, the geographic region and timeframe within which the study took place should be reported in the title or abstract.<br><br>RECORD 1.3: If linkage between databases was conducted for the study, this should be clearly stated in the title or abstract.	Page 1: Title page (case note review)<br><br>Page 1: Title page (England). The census periods have varied, but present within Appendix 1)<br><br>N/A No data linkage.
**Introduction**					
Background rationale	2	Explain the scientific background and rationale for the investigation being reported	Page 4-5: Introduction		
Objectives	3	State specific objectives, including any prespecified hypotheses	Page 5: Objectives		
**Methods**					
Study Design	4	Present key elements of study design early in the paper	Page 5: Study Design		
Setting	5	Describe the setting, locations, and relevant dates, including periods of recruitment, exposure, follow-up, and data collection	Page 5: Study Setting		

Participants	6	*(a) Cohort study* - Give the eligibility criteria, and the sources and methods of selection of participants. Describe methods of follow-up<br>*Case-control study* - Give the eligibility criteria, and the sources and methods of case ascertainment and control selection. Give the rationale for the choice of cases and controls<br>*Cross-sectional study* - Give the eligibility criteria, and the sources and methods of selection of participants<br><br>*(b) Cohort study* - For matched studies, give matching criteria and number of exposed and unexposed<br>*Case-control study* - For matched studies, give matching criteria and the number of controls per case	Page 5: Population	RECORD 6.1: The methods of study population selection (such as codes or algorithms used to identify subjects) should be listed in detail. If this is not possible, an explanation should be provided.<br><br>RECORD 6.2: Any validation studies of the codes or algorithms used to select the population should be referenced. If validation was conducted for this study and not published elsewhere, detailed methods and results should be provided.<br><br>RECORD 6.3: If the study involved linkage of databases, consider use of a flow diagram or other graphical display to demonstrate the data linkage process, including the number of individuals with linked data at each stage.	Page 5: Population. Enhanced criteria found in Appendix 2.<br><br>N/A<br><br>N/A
Variables	7	Clearly define all outcomes, exposures, predictors, potential confounders, and effect modifiers. Give diagnostic criteria, if applicable.	Page 9: Primary and Secondary Outcomes.	RECORD 7.1: A complete list of codes and algorithms used to classify exposures, outcomes, confounders, and effect modifiers should be provided. If these cannot be reported, an explanation should be provided.	Page 9: Primary and Secondary Oucomes. Avoidability ratings and Tier 1 and Tier 2 judgements found across Page 6-8.
Data sources/ measurement	8	For each variable of interest, give sources of data and details of methods of assessment (measurement).	Page 5: Data Screening and Page 6: Data Collection		

		Describe comparability of assessment methods if there is more than one group			
Bias	9	Describe any efforts to address potential sources of bias	Page 9: Bias		
Study size	10	Explain how the study size was arrived at	Page 10: Statistical analysis, advising on numerator and denominator.		
Quantitative variables	11	Explain how quantitative variables were handled in the analyses. If applicable, describe which groupings were chosen, and why	Page 10: Statistical analysis.		
Statistical methods	12	(a) Describe all statistical methods, including those used to control for confounding<br>(b) Describe any methods used to examine subgroups and interactions<br>(c) Explain how missing data were addressed<br>(d) *Cohort study* - If applicable, explain how loss to follow-up was addressed<br>*Case-control study* - If applicable, explain how matching of cases and controls was addressed<br>*Cross-sectional study* - If applicable, describe analytical methods taking account of sampling strategy<br>(e) Describe any sensitivity analyses	Page 10: Statistical analysis		
Data access and cleaning methods		..		RECORD 12.1: Authors should describe the extent to which the investigators had access to the database	Page 5: Study Setting

				population used to create the study population.<br><br>RECORD 12.2: Authors should provide information on the data cleaning methods used in the study.	(SystmOne) and Population.<br><br>Page 10: Statistical analysis.
Linkage		..		RECORD 12.3: State whether the study included person-level, institutional-level, or other data linkage across two or more databases. The methods of linkage and methods of linkage quality evaluation should be provided.	N/A no data linkage occurred.
**Results**					
Participants	13	(a) Report the numbers of individuals at each stage of the study (*e.g.*, numbers potentially eligible, examined for eligibility, confirmed eligible, included in the study, completing follow-up, and analysed)<br>(b) Give reasons for non-participation at each stage.<br>(c) Consider use of a flow diagram	Page 11: Descriptive statistics and flow chart present on Page 12.	RECORD 13.1: Describe in detail the selection of the persons included in the study (*i.e.*, study population selection) including filtering based on data quality, data availability and linkage. The selection of included persons can be described in the text and/or by means of the study flow diagram.	Page 11: Descriptive statistics and Page 12: Flow chart.
Descriptive data	14	(a) Give characteristics of study participants (*e.g.*, demographic, clinical, social) and information on exposures and potential confounders<br>(b) Indicate the number of participants with missing data for each variable of interest<br>(c) *Cohort study* - summarise follow-up time (*e.g.*, average and total amount)	Page 11: Descriptive statistics and flow chart present on Page 12.		

Outcome data	15	*Cohort study* - Report numbers of outcome events or summary measures over time<br>*Case-control study* - Report numbers in each exposure category, or summary measures of exposure<br>*Cross-sectional study* - Report numbers of outcome events or summary measures	Page 11: Descriptive statistics and flow chart present on Page 12.		
Main results	16	(a) Give unadjusted estimates and, if applicable, confounder-adjusted estimates and their precision (e.g., 95% confidence interval). Make clear which confounders were adjusted for and why they were included<br>(b) Report category boundaries when continuous variables were categorized<br>(c) If relevant, consider translating estimates of relative risk into absolute risk for a meaningful time period	Page 12: Overall rate of healthcare-associated harm,<br>Page 13: Rate of avoidable harm by gender<br>Page 14-15: Number and incidence of avoidable harm overall and in sub-groups<br>Page 16-18: Incident outcome and harm severity<br>Page 18-19: Avoidability ratings		
Other analyses	17	Report other analyses done—e.g., analyses of subgroups and interactions, and sensitivity analyses	Page 13: Interrater reliability<br>Page 20-21:Multi-level logistic regression analysis.		
**Discussion**					
Key results	18	Summarise key results with reference to study objectives	Page 22: Principle findings		
Limitations	19	Discuss limitations of the study, taking into account sources of potential bias or imprecision.	Page 22: Strengths and weaknesses of the study	RECORD 19.1: Discuss the implications of using data that were not created or collected to answer the specific research question(s). Include	Page 22: Strengths and limitations of the study

		Discuss both direction and magnitude of any potential bias		discussion of misclassification bias, unmeasured confounding, missing data, and changing eligibility over time, as they pertain to the study being reported.	
Interpretation	20	Give a cautious overall interpretation of results considering objectives, limitations, multiplicity of analyses, results from similar studies, and other relevant evidence	Page 23-24:Implications for policy and healthcare teams.		
Generalisability	21	Discuss the generalisability (external validity) of the study results	Page 23: Comparison with primary care		
**Other Information**					
Funding	22	Give the source of funding and the role of the funders for the present study and, if applicable, for the original study on which the present article is based	Page 25: Funding		
Accessibility of protocol, raw data, and programming code		..		RECORD 22.1: Authors should provide information on how to access any supplemental information such as the study protocol, raw data, or programming code.	Page 25: Data Sharing

*Reference: Benchimol EI, Smeeth L, Guttmann A, Harron K, Moher D, Petersen I, Sørensen HT, von Elm E, Langan SM, the RECORD Working Committee. The REporting of studies Conducted using Observational Routinely-collected health Data (RECORD) Statement. *PLoS Medicine* 2015; in press.

*Checklist is protected under Creative Commons Attribution (CC BY) license.
